# Fenton reaction facilitates organic nitrogen acquisition by an ectomycorrhizal fungus

**DOI:** 10.1111/nph.14971

**Published:** 2018-01-03

**Authors:** Michiel Op De Beeck, Carl Troein, Carsten Peterson, Per Persson, Anders Tunlid

**Affiliations:** ^1^ Department of Biology Microbial Ecology Group Lund University Ecology Building SE‐223 62 Lund Sweden; ^2^ Department of Astronomy and Theoretical Physics, Computational Biology and Biological Physics Lund University Sölvegatan 14A SE‐223 62 Lund Sweden; ^3^ Centre for Environmental and Climate Research (CEC) Lund University Ecology Building SE‐223 62 Lund Sweden

**Keywords:** Fenton reaction, nitrogen (N), *Paxillus involutus*, proteolysis, soil organic matter (SOM)

## Abstract

- Boreal trees rely on their ectomycorrhizal fungal symbionts to acquire growth‐limiting nutrients, such as nitrogen (N), which mainly occurs as proteins complexed in soil organic matter (SOM). The mechanisms for liberating this N are unclear as ectomycorrhizal fungi have lost many genes encoding lignocellulose‐degrading enzymes present in their saprotrophic ancestors. We hypothesized that hydroxyl radicals (^˙^ OH), produced by the ectomycorrhizal fungus *Paxillus involutus* during growth on SOM, are involved in liberating organic N.
- *Paxillus involutus* was grown for 7 d on N‐containing or N‐free substrates that represent major organic compounds of SOM. ^˙^ OH production, ammonium assimilation, and proteolytic activity were measured daily.
- ^˙^ OH production was strongly induced when *P. involutus* switched from ammonium to protein as the main N source. Extracellular proteolytic activity was initiated shortly after the oxidation. Oxidized protein substrates induced higher proteolytic activity than unmodified proteins. Dynamic modeling predicted that ^˙^ OH production occurs in a burst, regulated mainly by ammonium and ferric iron concentrations.
- We propose that the production of ^˙^ OH and extracellular proteolytic enzymes are regulated by similar nutritional signals. Oxidation works in concert with proteolysis, improving N liberation from proteins in SOM. Organic N mining by ectomycorrhizal fungi has, until now, only been attributed to proteolysis.

## Introduction

In boreal forests, tree growth is primarily limited by nutrient availability, in particular nitrogen (N) (Jarvis & Linder, 2000). Most N in boreal forest ecosystems occurs in the form of protein‐N (Friedel & Scheller, 2002; Nannipieri & Eldor, 2009). As boreal trees have a limited capacity to assimilate protein‐N directly (Näsholm *et al*., 2009), they strongly rely on their ectomycorrhizal (ECM) fungal symbionts to gain access to this form of N (Hobbie & Högberg, 2012). The utilization of proteins by fungi requires the degradation of these proteins into peptides and amino acids before cellular uptake. Extracellular proteolytic enzymes are critical for the degradation of proteins and are widespread in ECM fungi (Nygren *et al*., 2007). After being deposited in the soil, proteins form complexes with (poly)phenolic compounds, polysaccharides, and other degradation products of plants and microbial biopolymers present in soil organic matter (SOM) (Piccolo, 2001). This renders the complexed proteins less available for proteolytic degradation (Bending & Read, 1996). Hence, proteolytic enzymes are likely to work in concert with other degradation mechanisms to efficiently liberate organic N from SOM.

Recent analyses of ECM fungal genome sequences revealed that ECM fungi have lost many genes encoding lignocellulose‐degrading enzymes present in their saprotrophic ancestors, suggesting that ECM fungi have a reduced capacity to decompose SOM (Kohler *et al*., 2015) and consequently a reduced capacity to obtain N complexed in SOM (Pellitier & Zak, 2018). It has nevertheless been demonstrated, using laboratory‐scale experiments and spectroscopy, that the ECM fungus *Paxillus involutus* can oxidize lignocellulosic material in SOM extracts using a nonenzymatic mechanism involving hydroxyl radicals (^˙^OH) produced by the Fenton reaction (Fe^2+^ + H_2_O_2_ + H^+ ^→ Fe^3+^ + ^˙^OH + H_2_O), similar to the mechanism used by saprotrophic, wood‐decaying brown rot fungi (Rineau *et al*., 2012). A recent study has further demonstrated that the capacity to oxidize SOM is widespread among ECM fungi (Shah *et al*., 2016). In brown rot fungi, secreted metabolites are produced by the fungus that drive one‐electron reduction of Fe^3+^ and O_2_, generating the Fenton chemistry reagents Fe^2+^ and H_2_O_2_ (Hatakka & Hammel, 2010). One such Fe^3+^‐reducing metabolite, the diarylcyclopentenone involutin, is also secreted by *P. involutus* during SOM decomposition (Shah *et al*., 2015), but the source of H_2_O_2_ in *P. involutus* remains unknown.

In brown rot wood decomposers,^˙^OH initiate wood degradation by destabilizing the dense and strongly lignified wood cell walls (Kim *et al*., 2015). After the initial ^˙^OH attack, hydrolytic enzymes can penetrate the wood cell walls and liberate sugars, which serve as the main energy source for these saprotrophic fungi. Unlike brown rot fungi, ECM fungi do not obtain most of their metabolic carbon (C) from the substrate they grow on. Instead, photosynthetic sugars are provided by an ECM host plant (Nehls *et al*., 2010). It has therefore been suggested that ECM fungi that evolved from brown rot fungi (e.g. Boletales) have adapted the oxidative decomposition system from their saprotrophic ancestors to liberate N complexed in SOM (Lindahl & Tunlid, 2015). The aim of the current study was to investigate whether ^˙^OH are involved in N acquisition by the ECM fungus *P. involutus*.

## Materials and Methods

### Culture and growth conditions

*Paxillus involutus* (Batsch) Fr. strain ATCC 200175 (Manassas, VA, USA) was maintained on modified Fries medium (Fries, 1978). The medium contained 3.74 mM NH_4_Cl, 0.41 mM MgSO_4_·7H_2_O, 0.22 mM KH_2_PO_4_, 0.18 mM CaCl_2_·2H_2_O, 0.34 mM NaCl, 1.34 mM KCl, 0.24 mM H_3_BO_3_, 20 μM ZnSO_4_·7H_2_O, 5.01 μM CuSO_4_·5H_2_O, 50.29 μM MnSO_4_·H_2_O, 0.16 μM (NH_4_)_6_Mo_7_O_24_·7H_2_O, 73.99 μM FeCl_3_·6H_2_O, 33.30 mM d‐glucose, 55.51 μM myo‐inositol, 0.30 μM thiamine·HCl, 0.10 μM biotin, 0.59 μM pyridoxine, 0.27 μM riboflavin, 0.82 μM nicotinamide, 0.73 μM *p*‐aminobenzoic acid, and 0.46 μM Ca‐pantothenate. The pH of the medium was adjusted to 4.8 using HCl and KOH. Agar was added to 1% (w/v). Cultures were grown at 21°C in the dark.

### Experimental treatments

Cultures were grown on a monolayer of glass beads with a diameter of 4 mm (Rineau *et al*., 2012) in liquid Fries medium for 9 d. After day 9, the medium was removed and replaced with liquid Fries medium without NH_4_^+^ to induce N starvation (Shah *et al*., 2013). After 24 h, the starvation medium was removed and replaced with treatment medium (Table 1). In the treatment media, N‐containing and N‐free substrates representing major components of SOM were provided to the fungus to study how substrates of different chemical composition would affect the production of ^˙^OH. As it was observed that an induced production of ^˙^OH only occurred in media containing a protein (i.e. BSA), further experiments were conducted in the presence of BSA to elucidate the role of [Fe^3+^], [NH_4_^+^] and proteolytic activity for the ^˙^OH production (Table 1). All treatment media consisted of the base Fries medium supplemented with a high concentration of glucose (i.e. 33.30 mM d‐glucose), as it was previously shown that glucose is required by *P. involutus* to oxidize organic substrates (Rineau *et al*., 2013) and to ensure that the supply of readily available sugars did not become a limiting factor during the experiments. Iron was supplied to the treatment media in the oxidized form (i.e. Fe^3+^) to ensure that any Fe^2+^ detected in the growth media would result from reduction of Fe^3+^ by the fungal cultures. For the protease inhibition experiment, pepstatin A was added to a final concentration of 0.15 μM. All treatment media were filter‐sterilized. Cultures were grown on the treatment media for 7 d. All experiments were performed with three biological replicates.

**Table 1:** Fenton reaction inducing conditions tested

Organic compound/growth medium^a^	Organic compound concentration (mg l^−1^)	[N] (mM)^b^	[Fe^3+^] (μM)
No organic substrate	–	3.74 (NH_4_Cl)	74.0^c^
CMC	100.0	3.74 (NH_4_Cl)	74.0
Pectin	250.0	3.74 (NH_4_Cl)	74.0
Tannic acid	250.0	3.74 (NH_4_Cl)	74.0
COL	610.1	3.74 (NH_4_Cl) + 3.74 (COL)	74.0
PVP	436.5	3.74 (NH_4_Cl) + 3.74 (PVP)	74.0
BSA^d^	331.5	3.74 (NH_4_Cl) + 3.74 (BSA)	74.0
No organic substrate–low iron	–	3.74 (NH_4_Cl)	0.74
BSA–low iron^d^	331.5	3.74 (NH_4_Cl) + 3.74 (BSA)	0.74
BSA–NH_4_Cl spike day 1	331.5	3.74 (NH_4_Cl) + 3.74 (BSA) 1.87 (NH_4_Cl, added on day 1)	74.0
BSA–pepstatin A	331.5	3.74 (NH_4_Cl)	74.0
BSA–no NH_4_Cl	331.5	3.74 (BSA)	74.0

CMC, carboxymethyl cellulose; COL, chitosan oligosaccharide lactate; PVP, polyvinylpirrolidone.

a In all cases, the medium was a liquid nutrient Fries medium supplemented with nitrogen (N)‐containing or N‐free organic compounds representing the major components of soil organic matter. The N nutritional conditions and [Fe^3+^] were varied, as shown. *Paxillus involutus* cultures were incubated on the respective media for 7 d at 23°C in the dark.

b The N source is indicated in parentheses.

c In the medium with no organic substrate, Fe^3+^ hydrolyzed, aggregated, and was largely removed during the filtration step, with a resulting [Fe^3+^] of *c*. 18 μM.

d The BSA concentration was increased to 10 mg ml^−1^ for the experiments when the BSA carbonyl content was measured.

### Determination of [^˙^OH]

[^˙^OH] was determined by adding 100 μM (final concentration) coumarin to the growth media. Coumarin is a specific probe for the detection of ^˙^OH with low cytotoxicity (Liu *et al*., 2016). One of the reaction products formed after^˙^OH oxidation of coumarin is the fluorescent molecule umbelliferone. Umbelliferone concentration was measured in culture filtrates using an LS50B fluorescence spectrophotometer (*λ*_ex_ = 325 nm, *λ*_em_ = 455 nm; Perkin Elmer, Waltham, MA, USA). As the fluorescence of umbelliferone was found to be pH‐sensitive (data not shown), 755 μl of culture filtrate was acidified to pH 2 with 5 μl of 1 M HCl before fluorescence measurement. Umbelliferone concentration was determined relative to a standard curve constructed using 0–100 nM umbelliferone at pH 2. As controls, fluorescence was also determined in culture filtrates in the absence of coumarin, and for sterile treatment media with coumarin (data not shown).

### Determination of BSA carbonyl content

The coumarin probe used to detect ^˙^OH has a low molecular weight (146.15 g mol^−1^); it may be taken up by fungal cells and oxidized intracellularly. Secretion of oxidized coumarin could therefore affect the determination of extracellular [^˙^OH]. Hence, the oxidation of BSA was also evaluated by determining the amount of protein carbonyl groups; this approach is commonly used to assess oxygen radical‐mediated protein modifications (Levine *et al*., 1990). The carbonyl content of BSA was determined under conditions where the ^˙^OH production was either induced (i.e. in BSA growth medium) or not induced (i.e. BSA–low Fe) but the BSA concentration was increased to 10 mg ml^−1^ to obtain a sufficiently high BSA concentration for the assay. Protein carbonyl content was evaluated using a protein carbonyl content assay kit (Sigma Aldrich). BSA concentration was determined using the bicinchoninic acid assay kit (Sigma Aldrich). The carbonyl content of BSA was calculated as nmol carbonyl mg^–1^ protein.

### Determination of [Fe^2+^] and [NH_4_^+^]

[Fe^2+^] was determined using the ferrozine assay (Goodell *et al*., 2006). Briefly, 100 μl of culture filtrate was mixed with 1 ml of 0.1 M acetate buffer (pH 4.5) and 100 μl of 1% (w/v) ferrozine. Absorption at 562 nm was measured after 5 min of incubation at room temperature using an Ultrospec 3000 UV/visible light spectrophotometer (GE Healthcare, Chicago, IL, USA). [NH_4_^+^] were determined in culture filtrates daily, using flow injection analysis on a FIAstar 5000 Analyzer (Foss, Hillerød, Denmark).

### Determination of aspartic protease activity

It was previously demonstrated that almost all proteolytic activity in *P. involutus* during growth on BSA is associated with aspartic proteases (Shah *et al*., 2013). Protease activity was measured using the EnzChek protease assay kit (Thermo Fisher Scientific, Pittsburg, PA, USA). BODIPY·FL casein stock solutions (1 mg ml^−1^) were prepared in PBS; 10 μg ml^−1^ working solutions were prepared by diluting the stock solutions in citrate‐phosphate buffer (pH 3) containing 100 mM citrate monohydrate and 200 mM Na_2_HPO_4_·2H_2_O. The culture filtrate (100 μl) was mixed with an equal amount of BODIPY·FL casein working solution, and the mixture was incubated in the dark in a shaking incubator for 4 h at 37°C. After incubation, 100 μl of the mixture was transferred to a Greiner 96‐well black flat‐bottom plate (Kremsmünster, Austria) and sample fluorescence was measured using a FLUOstar Omega plate reader (*λ*_ex_ = 485 nm, *λ*_em_ = 520 nm, gain = 1733; BMG Labtech, Ortenberg, Germany). Aspartate protease activities were expressed as arbitrary units.

### Dynamic modeling of the main factors involved in regulating the Fenton reaction in *P. involutus*

The purpose of modeling was to formalize the hypotheses on how N nutritional conditions regulate the Fenton reaction in *P. involutus*, and to test and refine these hypotheses by attempting to fit the model to experimental data. A dynamic model describing the factors affecting the induction of hydroxyl radical (^˙^OH) production in the ECM fungus *P. involutus* was constructed. The model is a deterministic dynamic system; i.e. it is a set of ordinary differential equations that describe the development of a biological system in time, including the uptake of N and the reduction of Fe^3+^, considering the initial concentrations of, for example, NH_4_^+^, Fe^3+^, and BSA in the growth media. The mechanism whereby the fungus initiates the production of H_2_O_2_ and, thereby, oxidative degradation of BSA is central to the model. Model building, optimization and parameter fitting were performed using a C++ program as described previously (Fogelmark & Troein, 2014). Further details about model building and parameter fitting can be found in Supporting Information Methods S1.

### Statistical analyses

Statistical analyses were performed using R v.3.3.2 (R Core Team, 2016). Normal distributions of the residuals of models were checked with the Shapiro–Wilk test; the homoscedasticity of variances was analyzed using either the Bartlett's test or the Fligner–Killeen test. Depending on the distribution of the estimated parameters, either one‐way ANOVA or the Kruskal–Wallis rank sum test was used to check for significant differences in variances of parameters. Two‐by‐two comparisons were performed using either Tukey's honestly significant difference (HSD) test or pairwise Wilcoxon rank sum test, as indicated in the text. The significance threshold used for all statistical tests was *P *<* *0.01.

## Results

### Induction of ^˙^OH production by *P. involutus* cultures

**Figure 1:**
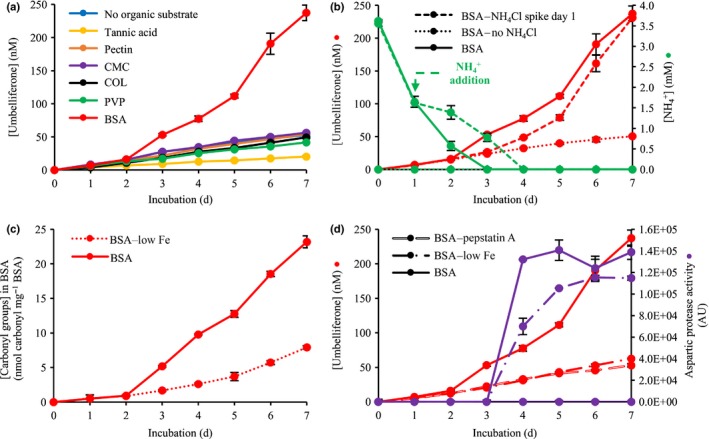
Extracellular hydroxyl radical (^˙^ OH) production by *Paxillus involutus*. The growth conditions are described in Table 1. (a) ^˙^ OH production in *P. involutus* cultures incubated with organic compounds in the presence of NH_4_^+^. ^˙^OH production was only induced in the presence of BSA. (b) ^˙^ OH production is induced when NH_4_^+^ becomes limiting. The addition of 1.87 mM NH_4_^+^ after the first day of incubation delayed the NH_4_^+^ depletion by 1 d; the induction of ^˙^ OH production was consequently also delayed by 1 d. No induction of ^˙^ OH production was seen in the BSA growth medium in the absence of NH_4_^+^. (c) Production of ^˙^ OH leads to the oxidation of BSA, resulting in an increased carbonyl content in BSA starting from day 3 of the incubation. The BSA concentration was increased to 10 mg ml^−1^ for the determination of the BSA carbonyl content. (d) Aspartic protease activity is induced 1 d after the induction of ^˙^ OH production. Aspartic protease activity was more pronounced in the presence of an oxidized protein substrate than in nonoxidized protein substrate. The addition of pepstatin A to the growth medium efficiently blocked extracellular aspartic protease activity and prevented the induction of ^˙^ OH production. Data points represent averages of three biological replicates (*n *=* *3). Error bars, ± SD. In (a), (b) and (d), the same data for umbelliferone concentration for the BSA growth medium are shown. CMC, carboxymethyl cellulose; COL, chitosan oligosaccharide lactate; PVP, polyvinylpirrolidone; AU, arbitrary units.

In all media, except for that containing both protein (i.e. BSA) and NH_4_^+^, *P. involutus* produced low but steady amounts of ^˙^OH (Fig. 1a). Presence of BSA in the growth medium was associated with significantly induced ^˙^OH production compared with BSA‐free media (Tukey's HSD test, *P *<* *0.01, for days 3–7 for all pairwise comparisons). Induced ^˙^OH production was observed starting from day 3 of the incubation period and coincided with a depletion of NH_4_^+^ in the growth medium (Fig. 1b). When an additional portion of NH_4_^+^ was spiked into the BSA growth medium after the first day of incubation (BSA–NH_4_Cl spike day 1), NH_4_^+^ was depleted by day 4 of the incubation period instead of day 3, and the induction of ^˙^OH production was also delayed by 1 d (Fig. 1b). When no NH_4_^+^ was provided in the BSA growth medium (BSA–no NH_4_Cl), no induction of ^˙^OH production was observed (Fig. 1b). These results clearly indicate that the induction of ^˙^OH production by *P. involutus* is regulated by a combination of limiting [NH_4_^+^] and the presence of protein as an alternative N source.

### Oxidation of protein substrate by *P. involutus* cultures

The increase in BSA carbonyl content (Fig. 1c) showed a very similar pattern to the increase in [^˙^OH] determined using the coumarin probe (Fig. 1d), and indeed the two measurements were strongly correlated (Pearson's product–moment correlation, *r* ^2^ = 0.99, *P *<* *0.01). Hence, we show that *P. involutus* produced ^˙^OH extracellularly and that these radicals oxidized the BSA protein in the growth medium.

### Aspartic proteolytic activity of *P. involutus* cultures

The major extracellular proteolytic activity of *P. involutus* during growth on BSA‐containing medium is associated with aspartic proteases (Shah *et al*., 2013). Indeed, the protease activity in the BSA growth medium was almost completely abolished by the addition of the aspartic protease inhibitor pepstatin A (BSA–pepstatin A in Fig. 1d). Aspartic protease inhibition, however, also prevented the induction of ^˙^OH production (Fig. 1d). This observation supports the notion that a suitable protein substrate must be detected by *P. involutus* for the induction of ^˙^OH production to occur.

Aspartic proteases produced by *P. involutus* during growth on BSA‐containing medium have an optimal activity at acidic pH values, with proteolytic activity sharply dropping at pH 4 and above (Shah *et al*., 2013). In our experiments, pH values of the growth media decreased rapidly from 4.8 to below 3 after the first day of incubation (Fig. S1), probably as a result of uptake of NH_4_^+^ from the growth medium. Hence, the pH of the growth media should not be an inhibiting factor for aspartic protease activity in our experiments. The induction of aspartic protease activity of *P. involutus* occurred on day 4, 1 d after the induction of ^˙^OH production (Fig. 1d). Spiking an additional amount of NH_4_^+^ into the BSA growth medium after the first day of incubation (BSA–NH_4_Cl spike day 1) also delayed the induction of the aspartic protease activity by 1 d (Fig. S2). These observations strongly suggest that the aspartic protease activity and production of ^˙^OH in *P. involutus* are induced by similar nutritional signals.

Lowering the [Fe^3+^] in the BSA growth medium (BSA–low iron) prevented the induction of ^˙^OH production and resulted in a reduced proteolytic activity compared with the BSA growth medium (Tukey's HSD test, *P *<* *0.01, for incubation days 4, 5, and 7) (Fig. 1d).

### Iron reduction by *P. involutus* cultures

Similar [Fe^2+^] values were detected in media containing BSA, carboxymethyl cellulose (CMC), chitosan oligosaccharide lactate (COL) or pectin. Under these conditions, all iron in the growth media was in the reduced form by day 7 (Fig. S3a). Similar [Fe^2+^] were detected in the BSA growth medium and ‘BSA–NH_4_Cl spike day 1’ growth medium. Adding pepstatin A to the BSA growth medium (BSA–pepstatin A) resulted in slightly, yet significantly, reduced [Fe^2+^] (Tukey's HSD test, *P *<* *0.01, for incubation days 1–7; Fig. S3b). However, in the BSA growth medium lacking NH_4_^+^ (BSA–no NH_4_Cl), the resultant [Fe^2+^] was four times lower on incubation day 7 (Fig. S3b). Hence, it appears that some NH_4_^+^ must be available to the fungus to drive the metabolic pathways that produce iron‐reducing molecules in *P. involutus*. Based on these observations, it may be further concluded that a high [Fe^2+^] is a prerequisite for, but does not necessarily result in, an induction of ^˙^OH production.

### Dynamic modeling of the regulation of the Fenton reaction

**Figure 2:**
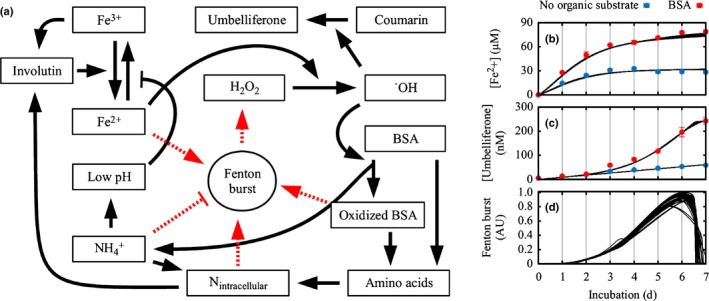
Dynamic modeling of the induction of the Fenton reaction in *Paxillus involutus*. (a) Graphical representation of the model network. Arrows, positive interactions (conversion, induction); arrows with bars, negative interactions (inhibition) between model components (boxes); dashed red lines, the regulation of the Fenton reaction through H_2_O_2_ production. (b, c) Timescales for the development of [Fe^2+^] (b) and umbelliferone concentration (c), according to the experiments (circles) and as predicted by the model (black lines), in BSA medium (red) or in a medium lacking an organic substrate (blue). Data points represent averages of three biological replicates (*n *=* *3). Error bars, ± SD. (d) Modeled burst of Fenton activity in a medium with BSA. The model is represented by an ensemble of 80 fitted parameter sets, as discussed in Supporting Information Methods S1. The Fenton burst curves were normalized to a maximum of 1 across experiments for each parameter set. AU, arbitrary units.

Next, a mathematical model was constructed to predict the regulation of the Fenton reaction by *P. involutus* in response to the availability of NH_4_^+^‐N and/or protein‐N. The model was based on three interconnected parts: an input part concerning NH_4_^+^, pH, and Fe^2+^/Fe^3+^ redox cycling; a central part, where the intracellular N‐pool interacts with the different input parts to regulate the Fenton reaction (i.e. H_2_O_2_ production); and, finally, an output part which encompasses the resulting ^˙^OH, and the oxidation and proteolysis of BSA (Fig. 2a). Multiple different model configurations were explored and rejected, either on account of inconsistency with the experimental data or for being too complex and thus prone to overfitting and difficult to interpret (Methods S1). Comparisons of the modeled and experimental data are shown in Fig. 2(b*,*d) and in Fig. S4; the fitted parameter values are presented in Fig. S5.

The main regulator of the Fenton chemistry in the model was the [NH_4_^+^], directly inhibiting the production of H_2_O_2_ but indirectly enabling the Fenton reaction via a putative intracellular N‐pool (denoted as N_intracellular_), representing, for example, intracellular amino acid concentrations. This mechanism allowed the Fenton reaction to be induced as a burst for a few days, in response to decreasing [NH_4_^+^] when BSA was available. The initial models predicted that oxidative degradation was induced much later and more abruptly than was suggested by empirical data. This discrepancy could only be solved by including a positive feedback mechanism between BSA oxidation and H_2_O_2_ production, with the [NH_4_^+^] controlling the time development of the Fenton burst. Furthermore, the model was able to account for the behavior of [Fe^2+^] and its low values in the absence of NH_4_^+^ only when the iron reduction was limited by the intracellular N pool and [Fe^3+^]. The model further suggested that the [Fe^2+^] tightly regulates the activity of the Fenton system. The model‐predicted outcomes accurately reflected the experimental data, indicating that the major signals involved in the regulation of the Fenton reaction in *P. involutus* were indeed identified in the current study.

## Discussion

A previous study on the involvement of the Fenton reaction in SOM degradation by *P. involutus* showed that glucose must be present in the culture medium for the fungus to oxidize SOM (Rineau *et al*., 2013). In the current study, by conducting more detailed analyses using a synthetic medium supplemented with various representative organic compounds, we identified three factors, in addition to sufficiently high concentrations of glucose, that are required for the induction of ^˙^OH production in *P. involutus*: sufficient iron, in its reduced form; protein, accessible to the ECM fungus; and limiting [NH_4_^+^]. The empirical data hence suggest that the Fenton reaction is induced when *P. involutus* switches from NH_4_^+^ to protein as its main N source. Furthermore, we demonstrated that the Fenton mechanism operates in sequence with an extracellular proteolytic system, i.e. with oxidation of a protein substrate preceding proteolysis. Dynamic modeling provided further insights into the regulation of the Fenton‐based oxidative system. It predicted that the production of the oxidative ^˙^OH occurs in a burst, and that [NH_4_^+^] and [Fe^2+^] are the key factors controlling ^˙^OH production in *P. involutus*. The model also predicted that the timing of the onset of the Fenton burst is controlled by a positive feedback mechanism, with the fungus sensing and responding to the degradation products of an oxidized protein substrate.

We observed similar [Fe^2+^] in growth media containing BSA, CMC, COL, and pectin (Fig. S3a). In all cases, most of the Fe^3+^ occurred in the reduced Fe^2+^ form after 7 d of incubation. It is currently unclear why most of the iron was reduced by *P. involutus*, but low pH values in the culture media (reaching a pH of 2.4 after 7 d of incubation in most treatments; Fig. S1) could have stabilized the iron in its reduced form. If an extensive iron reduction were also to occur in forest soils, this could have implications for soil formation and potentially explain why in typical podzol soils under forest ecosystems a pronounced illuvial horizon depleted in iron can be observed. Nevertheless, an induction of ^˙^OH production was observed after NH_4_^+^ became depleted only in the BSA growth medium (Fig. 1a). Hence, high [Fe^2+^] does not necessarily result in an appreciable production of ^˙^OH in *P. involutus*. It is likely that iron reduction and H_2_O_2_ production are not a consequence of a single mechanism as, for example, is the case in the brown rot fungus *Gloeophyllum trabeum*. In *G. trabeum*, the secondary metabolite 2,5‐dimethoxy‐1,4‐hydroquinone is capable of both reducing Fe^3+^ and producing H_2_O_2_ (Jensen *et al*., 2001). By contrast, the major iron reductant produced by *P. involutus* (i.e. involutin) does not participate in the production of H_2_O_2_ (Shah *et al*., 2015). Although the mechanism of H_2_O_2_ generation in *P. involutus* is currently unknown, it might involve the activity of specific oxidases, including copper radical oxidases, members of the glucose‐methanol‐choline oxidoreductase family and (*S*)‐2‐hydroxy‐acid oxidases (EC 1.1.3.15) (Levasseur *et al*., 2013). Genes encoding such enzymes are indeed expressed by *P. involutus* during SOM oxidation (Shah *et al*., 2016).

As has been observed in other fungi (Wong *et al*., 2008; Zaman *et al*., 2008), *P. involutus* uses NH_4_^+^ before using an alternative N source, i.e. BSA. When the preferential N source NH_4_^+^ is present, genes required for the degradation of BSA are most likely repressed by N catabolite repression (NCR). This mechanism has been extensively studied in many fungi. During NCR, the presence of a preferred N source (typically, NH_4_^+^) represses the transcription of genes involved in the scavenging and metabolizing of alternative N sources (Wong *et al*., 2008; Zaman *et al*., 2008). In *Saccharomyces cerevisiae*, NH_4_^+^ limitation is sensed through the intracellular concentration of glutamine and/or glutamate, which are synthesized from assimilated NH_4_^+^ by the conserved enzymes glutamate dehydrogenase (GDH), glutamine oxoglutarate aminotransferase (GOGAT), and glutamine synthetase (GS) (Zaman *et al*., 2008). The corresponding components of an NH_4_^+^‐sensing system have been characterized in the ECM fungus *Hebeloma cylindrosporium* (Javelle *et al*., 2003), and homologs of GDH and GS‐GOGAT have been found in *P. involutus* (Shah *et al*., 2013).

Dynamic modeling of the Fenton mechanism suggests that the induction of ^˙^OH production in *P. involutus* is not only regulated by NCR but also by a positive feedback mechanism involving the sensing of protein degradation products released by extracellular proteases. In the absence of such a positive feedback mechanism, the cells would have to possess a precise mechanism for detecting low intracellular concentrations of glutamine/glutamate. The presence of such a mechanism is unlikely, as studies with *S*. *cerevisiae* have suggested that the NCR pathways respond gradually to N depletion (Zaman *et al*., 2008). The amino acids released by extracellular proteolytic enzymes of *P. involutus* are presumably sensed by intracellular mechanisms, as extracellular amino acid sensing mechanisms have not been described in any filamentous fungus (Van Dijck *et al*., 2017). Although the amino acid sensing mechanisms activated in *P. involutus* are not known, two conserved systems for sensing intracellular amino acid concentrations are known to operate in most eukaryotes: the general amino acid control pathway and the target of rapamycin kinase signaling pathway (Chantranupong *et al*., 2015).

During prolonged periods of N starvation in *S. cerevisiae*, N‐deprived cells recycle intracellular proteins via the autophagy pathway to generate amino acids required for protein synthesis (Onodera & Ohsumi, 2005). Genes encoding the conserved components of the autophagy pathway have been identified in the genome of *P. involutus* (Ellström *et al*., 2015), and it is therefore likely that this fungus also initiates an autophagy response during prolonged periods of N starvation. *P. involutus* cells grew very poorly in the ‘BSA–no NH_4_Cl’ growth medium in the current study. Moreover, four times less Fe^3+^ was reduced under these conditions than during growth in the presence of high [NH_4_^+^], with no induction of ^˙^OH production. These observations suggest an impaired fungal metabolic capacity to produce iron‐reducing molecules and ^˙^OH in the absence of a preferred N source. Abuzinadah & Read (1986) obtained high *P. involutus* biomass yields after 60 d of growth on BSA as a sole N source; this placed *P. involutus* in the group of so‐called ‘protein fungi’, in contrast to other ECM fungi which produced little or no biomass after 60 d of growth on BSA as a sole N source, classifying them as ‘nonprotein fungi’. In the current study, low protease activity was detected starting after day 4 of growth in the ‘BSA–no NH_4_Cl’ growth medium (Fig. S6). We conclude that proteolytic enzymes are active under N‐limiting conditions, but the degradation of protein is probably more efficient under conditions favoring ^˙^OH production.

^˙^OH may be involved in organic N acquisition by ECM fungi in various ways. First, ^˙^OH oxidation of proteins may render the proteins more susceptible to proteolytic degradation. Increased proteolysis following metal‐catalyzed radical oxidation of proteins has been demonstrated for a wide range of proteolytic enzymes and proteins (Zhang *et al*., 2016). In the current study, aspartic protease activity was appreciably higher once BSA had been oxidized by ^˙^OH (BSA growth medium) than when BSA was less oxidized (BSA–low Fe; Fig. 1d). The observed 1 d delay between the induction of ^˙^OH production and the induction of proteolytic activity could serve to oxidatively ‘mark’ BSA for further hydrolytic degradation. Interestingly, modeling predicted at least a hundred‐fold increase in proteolysis rate once BSA had been oxidized, suggesting that an enhanced susceptibility of oxidized proteins to proteolytic degradation might be important for the extracellular degradation of proteins by *P. involutus*. A similar temporal separation of the hydrolytic and oxidative degradation of intact wood by ^˙^OH has been observed in the brown rot wood decomposer *Postia placenta* (Schilling *et al*., 2013). Such a delay between the production of hydrolytic enzymes and ^˙^OH generation may also protect the hydrolytic enzymes from becoming oxidized themselves (Schilling *et al*., 2013).

Second, amine groups of proteins, peptides, and amino acids are preferred targets of ^˙^OH produced by the Fenton reaction (Stadtman, 1993). Radical oxidation of amine groups results in the formation of carbonyl groups and the liberation of NH_4_^+^ (Stadtman, 1990). The formation of carbonyl groups correlated well with the oxidation of the coumarin radical probe in the current study (Fig. 1b,c), suggesting that the protein substrate was indeed extracellularly oxidized by ^˙^OH. Hence, it is likely that the induction of ^˙^OH production resulted in the release of additional NH_4_^+^ ions from BSA, which could help to alleviate N starvation. In agreement with this hypothesis, it has been reported that genes encoding NH_4_^+^ transporters are up‐regulated in *P. involutus* during the assimilation of organic N, including BSA (Shah *et al*., 2013). Furthermore, ECM fungi do not assimilate all naturally occurring amino acids with the same efficiency. Several authors studied *P. involutus* biomass yields after growth on a single amino acid as the sole N source. Of the 13 amino acids that have been tested, only three (arginine, glutamine, and glutamic acid) were able to sustain good growth of *P. involutus* (Laiho, 1970; Lundeberg, 1970; Finlay *et al*., 1992; Chalot & Brun, 1998; Sarjala, 1999). If only a small proportion of the proteolysis reaction products is used to sustain cellular growth, the production of extracellular proteolytic enzymes would prove to be a costly process. ^˙^OH could directly liberate NH_4_^+^ from a number of amino acids and peptides, which would otherwise not serve as sources of metabolic N. Hence, the Fenton reaction might broaden the spectrum of substrates from which ECM fungi derive metabolic N.

Third, ECM fungi may encounter proteins in a relatively pure form in SOM only when these proteins are recently deposited. Proteins undergo complexation with (poly)phenolic compounds in SOM (Vanburen & Robinson, 1969). The capacity to degrade tannin–protein complexes by mycorrhizal fungi has been studied, and Bending & Read (1996) concluded that *P. involutus* is unable to degrade such complexes. However, the potential involvement of ^˙^OH in the oxidative degradation of (poly)phenol–protein complexes by ECM fungi has not yet been considered. During brown rot wood decomposition, ^˙^OH oxidation of the highly lignified wood cell walls is suggested to facilitate the penetration of hydrolytic enzymes into the wood tissue (Kim *et al*., 2015). In a very similar manner, the liberation of protein‐N complexed in SOM may be a two‐step process, with oxidation facilitating proteolytic degradation.

It should be noted that the observations made in the current study were done in a pure culture system and it remains to be shown whether the regulation and production of ^˙^OH by the factors identified in the current study hold true in natural forest ecosystems and when the fungus grows in symbiosis with its host plant. Owing to the large difficulties in measuring ^˙^OH production in complex systems like soils, the activity of this process might be inferred from analyzing the expression levels of genes encoding enzymes involved in synthesizing the Fenton chemistry reactants. We have previously identified synthetases that are involved in the biosynthesis of the Fe^3+^‐reductant involutin (Braesel *et al*., 2015). Knowing the precise nutritional signals that regulate the induction of ^˙^OH production opens up new possibilities for characterizing the molecular components of the Fenton‐based protein degradation mechanism in ECM fungi. Such information is needed to examine the expression of nonenzymatic Fenton reactions using transcriptional analyses.

Based on the results described in the current study, it is clear that a switch from NH_4_^+^ to protein as the main N source induces the production of ^˙^OH in *P. involutus*. Furthermore, proteolytic activity is enhanced after oxidation of the protein substrate. Hence, the Fenton reaction could prove to be an important component of the N‐acquisition machinery of ECM fungi. A dual oxidative‐proteolytic system may be more efficient than a proteolytic system alone for releasing N from proteins complexed in SOM aggregates. Hence, our findings also suggest that the ancestral decay mechanisms used by brown rot fungi for releasing C from wood tissues have been adapted by ECM fungi that evolved from brown rot ancestors to scavenge for organic N complexed in SOM.

## Author contributions

All authors contributed to the writing of the manuscript; M.O.D.B., P.P. and A.T. designed the experiments; M.O.D.B. conducted the experiments; C.T. and C.P. designed and tested the dynamic model. M.O.D.B. and C.T. contributed equally to this work.

## Supporting information

Please note: Wiley Blackwell are not responsible for the content or functionality of any Supporting Information supplied by the authors. Any queries (other than missing material) should be directed to the *New Phytologist* Central Office.

**Fig. S1** Changes in pH values in growth experiments summarized in Table 1.
**Fig. S2** NH_4_^+^ addition delays both the induction of ^•^OH production and aspartic protease activity in *Paxillus involutus* cultures.
**Fig. S3** Changes in [Fe^2+^] in growth experiments summarized in Table 1.
**Fig. S4** Model fits and predictions under different experimental growth conditions.
**Fig. S5** Distributions of fitted model parameter values.
**Fig. S6** Aspartic protease activity is low in the absence of NH_4_Cl.
**Methods S1** Detailed description of dynamic model building, parameter fitting, and sensitivity analyses.
