# Supplementary material for: Fenton reaction facilitates organic nitrogen acquisition by an ectomycorrhizal fungus
Source: New Phytol. 2018 Jan 3;218(1):335–43. doi: 10.1111/nph.14971 (PMC5873446; doi:10.1111/nph.14971)
Supplement: Supplementary file 1 — Fig. S1 Changes in pH values in growth experiments summarized in Table 1. Fig. S2 NH4 + addition delays both the induction of •OH production and aspartic protease activity in Paxillus involutus cultures. Fig. S3 Changes in [Fe2+] in growth experiments summarized in Table 1. Fig. S4 Model fits and predictions under different experimental growth conditions. Fig. S5 Distributions of fitted model parameter values. Fig. S6 Aspartic protease activity is low in the absence of NH4Cl. Methods S1 Detailed description of dynamic model building, parameter fitting, and sensitivity analyses. [file NPH-218-335-s001.pdf]

New Phytologist **Supporting Information**

Article title: Fenton reaction facilitates organic nitrogen acquisition by an ectomycorrhizal fungus.
Authors: Michiel Op De Beeck, Carl Troein, Carsten Peterson, Per Persson, and Anders Tunlid.
Article acceptance date: 27 November 2017

The following Supporting Information is available for this article:

Fig. S1 Changes in pH values in growth experiments summarized in Table 1. pH values in culture filtrates of *Paxillus involutus* cultures grown for 7 d under different N-nutritional conditions are shown. pH values drop rapidly in the growth media during incubation with *Paxillus involutus*, but to a lesser extent when no NH_4Cl was provided. (a) Cultures were grown on different N-containing or N-free organic compounds in the presence of NH_4Cl. (b) Cultures were grown on different variations of the BSA growth medium as indicated. Data points represent averages of three biological replicates (n = 3). Error bars represent s.d. BSA, bovine serum albumin; CMC, carboxymethyl cellulose; COL, chitosan oligosaccharide lactate; PVP, polyvinylpirrolidone.

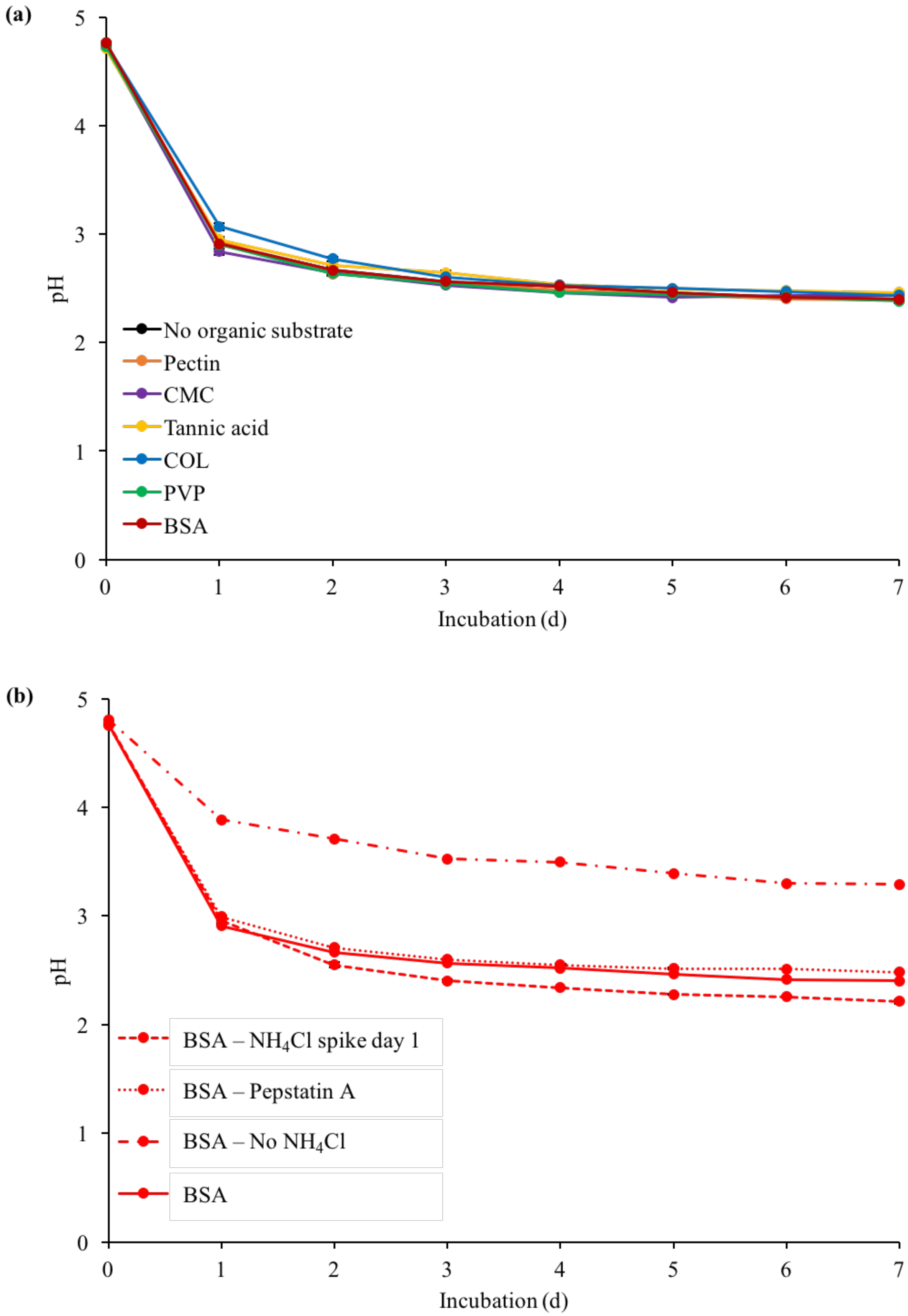

(a)
pH
No organic substrate
Pectin
CMC
Tannic acid
COL
PVP
BSA
Incubation (d)
(b)
pH
BSA – NH_4Cl spike day 1
BSA – Pepstatin A
BSA – No NH_4Cl
BSA
Incubation (d)

Fig. S2 NH_4^+ addition delays both the induction of $^{\bullet}OH$ production and aspartic protease activity in *Paxillus involutus* cultures. The induction of $^{\bullet}OH$ production was separated from the induction of aspartic protease activity by 1 d. They were both delayed by 1 d after an additional amount of NH_4Cl was spiked-in after the first day of incubation. Data points represent averages of three biological replicates (n = 3). Error bars represent s.d.

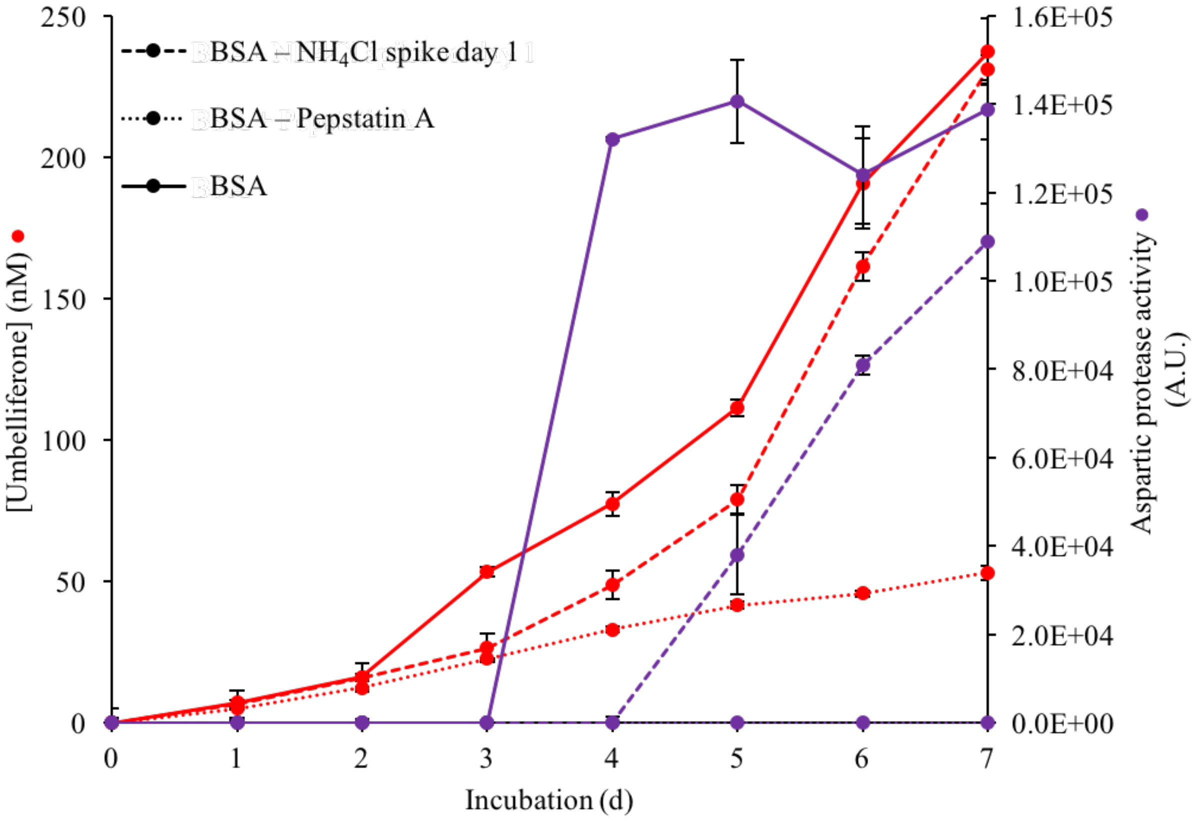

Fig. S3 Changes in [Fe^{2+}] in growth experiments summarized in Table 1. [Fe^{2+}] in culture filtrates of *Paxillus involutus* cultures grown for 7 d under different N-nutritional conditions are shown. (a) Cultures were grown on different N-containing or N-free organic compounds in the presence of NH_4Cl. The organic compounds were selected to represent major compounds present in soil organic matter. (b) Cultures were grown on different variations of the BSA growth medium as indicated. Data points represent averages of three biological replicates (n = 3). Error bars represent s.d. BSA, bovine serum albumin; CMC, carboxymethyl cellulose; COL, chitosan oligosaccharide lactate; PVP, polyvinylpirrolidone.

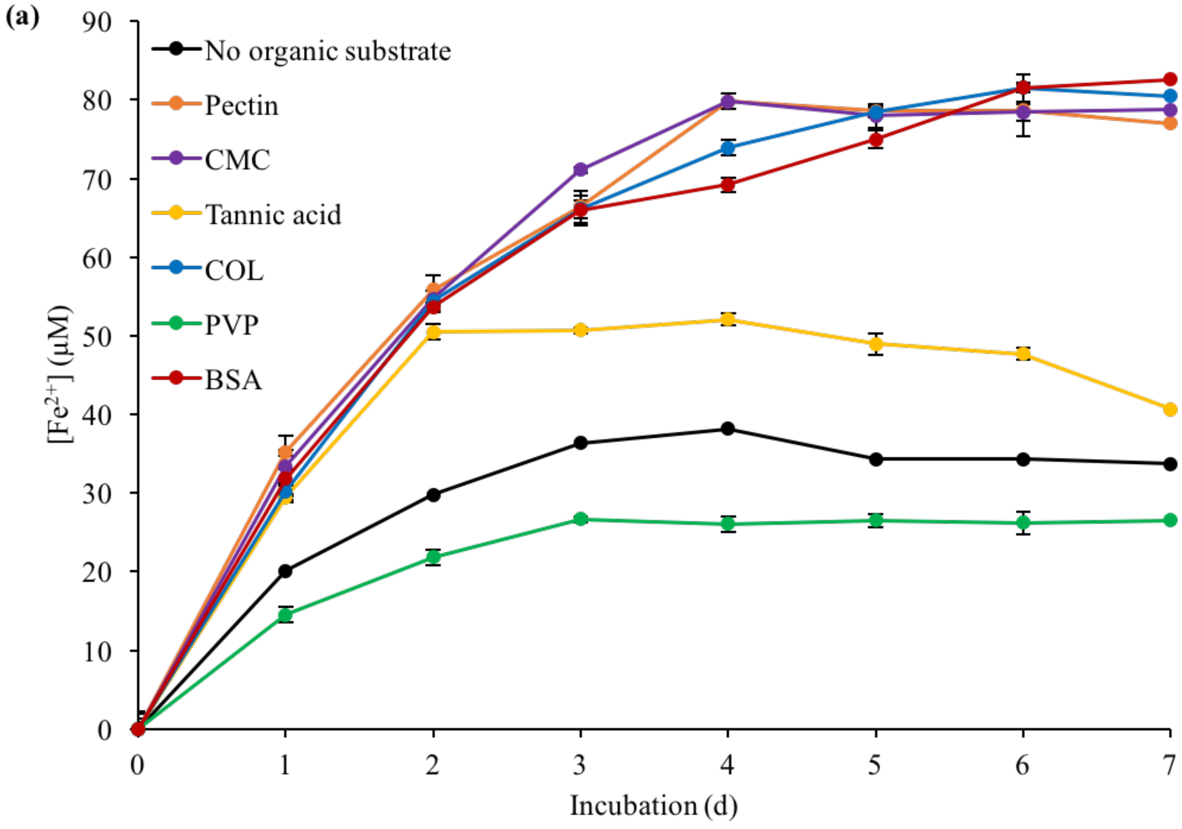

(b)

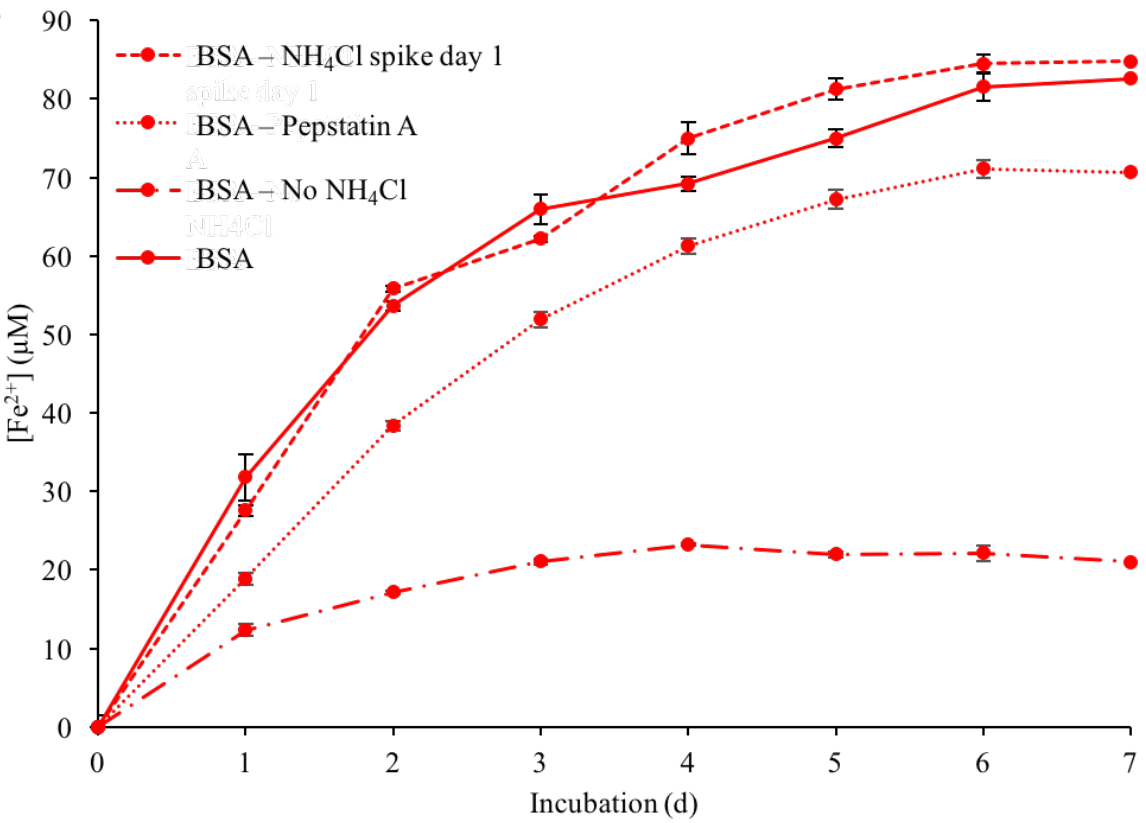

BSA – NH_4Cl spike day 1
BSA – Pepstatin A
BSA – No NH_4Cl
BSA
$[Fe^{2+}]$ (µM)
0
10
20
30
40
50
60
70
80
90
0
1
2
3
4
5
6
7
Incubation (d)

Fig. S4 Model fits and predictions under different experimental growth conditions. Comparisons of experimental data (circles) and model simulations (lines) for three types of data are shown: $[H^+]$ (top row), $[NH_4^+]$ (second row) and [umbelliferone] (third row). In addition, predictions of the Fenton burst regulating $^{\bullet}OH$ production are shown (bottom row). The columns represent six different growth conditions as indicated. Note the use of two different y-scales for different growth conditions. The model output is represented by one curve for each of 80 individually fitted parameter sets as described in Supporting Information Methods S1. Data points represent individual biological replicates. The Fenton burst curves were normalized to a maximum of 1 across the experiments for each parameter set.

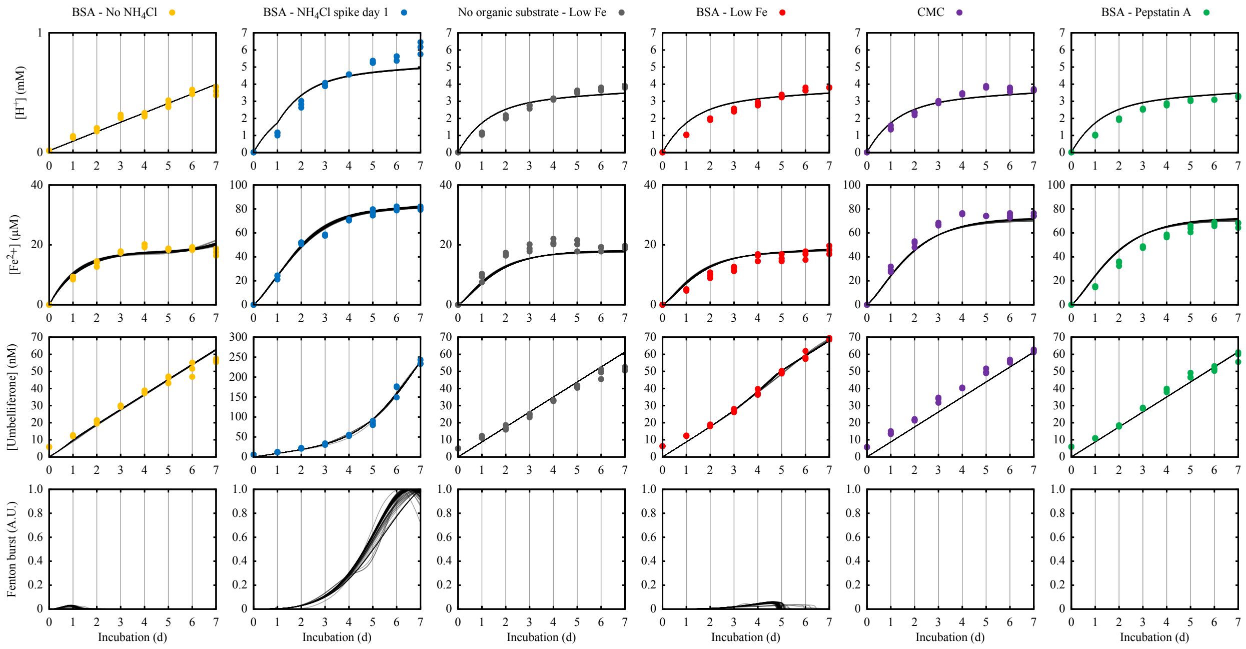

Fig. S5 Distributions of fitted model parameter values. Values of the 27 model parameters were fitted to the experimental data, as described in Supporting Information Methods S1 (the parameter values are defined therein). For each parameter, its values in the 80 individually fitted parameter sets were sorted and plotted against rank to show its distribution. Some parameters stayed within a narrow range, some were weakly constrained and spanned many orders of magnitude, while some reached the limits set based on various physical considerations. The y-scales are either linear or logarithmic as needed.

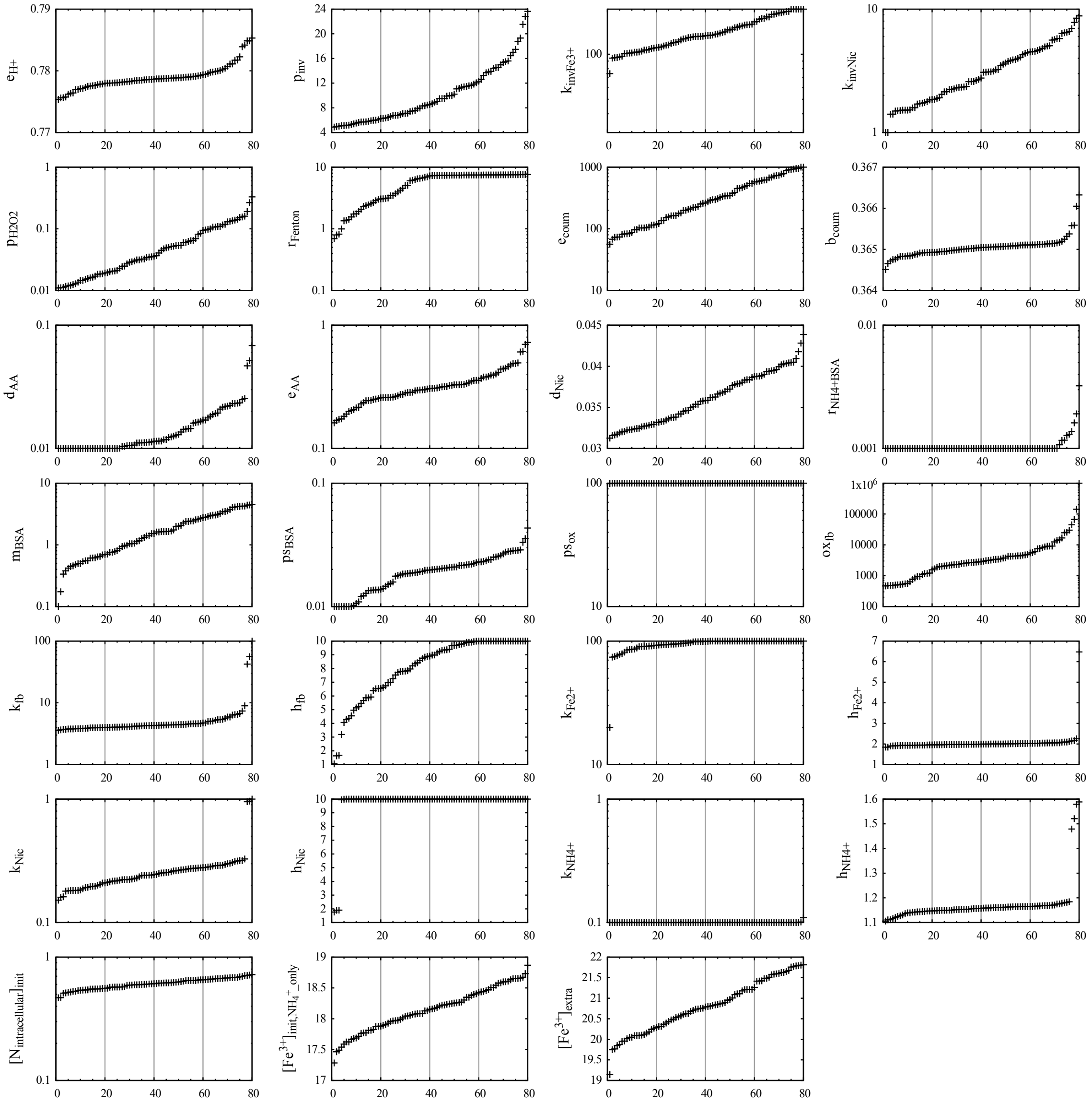

Fig. S6 Aspartic protease activity is low in the absence of NH_4Cl. Aspartic protease activity measured in culture filtrates of *Paxillus involutus* cultures grown for 7 d under different N-nutritional conditions is shown. A much lower aspartic protease activity was noted when *P. involutus* was grown on BSA as the sole N source (BSA - No NH_4Cl) than during growth on BSA supplemented with NH_4Cl (BSA growth medium). Details of the nutritional conditions for each treatment are summarized in Table 1. Data points represent averages of three biological replicates (n = 3). Error bars represent s.d.

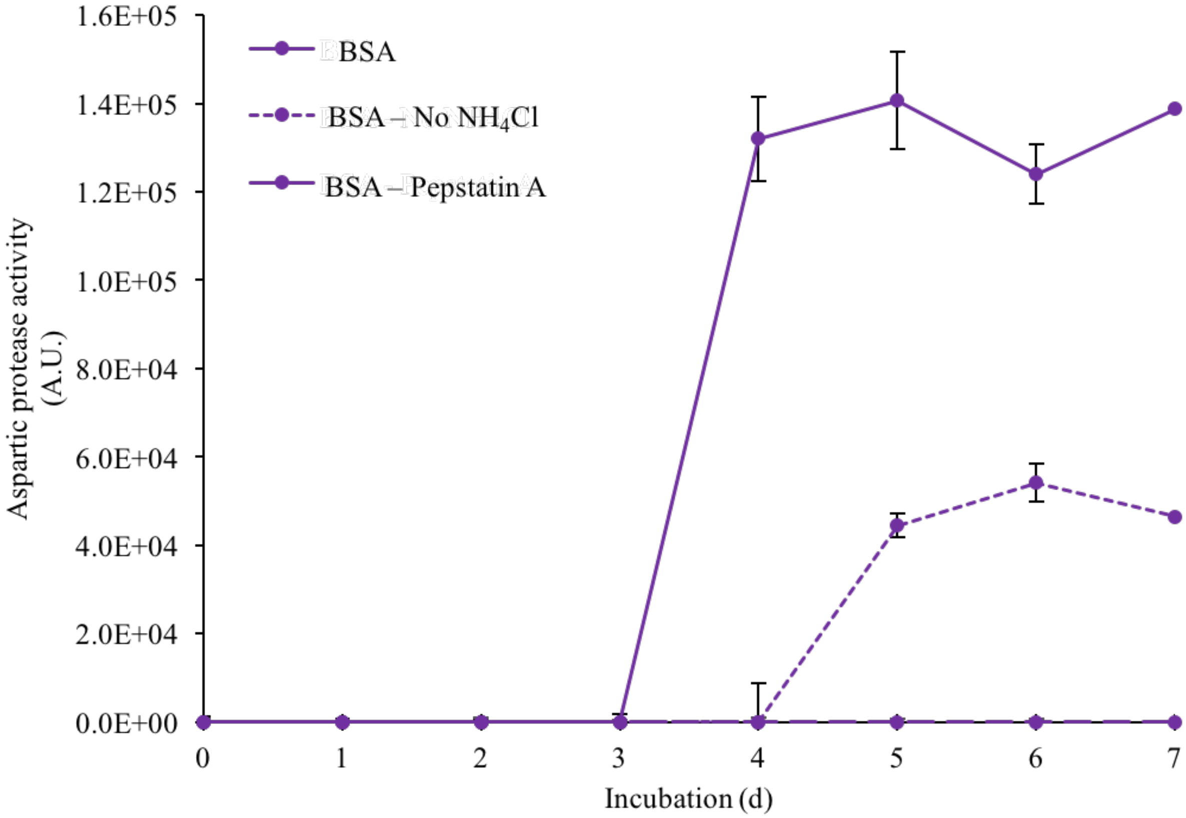

Methods S1 Modeling

The final model consists of a system of 14 ordinary differential equations that describe the development over time of the variables shown in boxes in Fig. 2a, with two exceptions: (1) the [coumarin] was assumed to be virtually constant throughout experiments; (2) $^{\bullet}$OH are extremely short-lived (He *et al.*, 2016) and were eliminated from the equations. The equations for the remaining 11 variables are as follows:

$$radicals = r_{\mathrm{Fenton}}[\mathrm{Fe^{2+}}][\mathrm{H_2O_2}]$$

$$\frac{d}{dt}[\mathrm{NH_4^+}] = -u_{\mathrm{NH4+}}[\mathrm{NH_4^+}] + r_{\mathrm{NH4+BSA}}\,radicals[\mathrm{BSA}]$$

$$\frac{d}{dt}[\mathrm{H^+}] = b_{\mathrm{H+}} + e_{\mathrm{H+}}u_{\mathrm{NH4+}}[\mathrm{NH_4^+}]$$

$$o_{\mathrm{Fe2+}}([\mathrm{H^+}]) = \frac{6\cdot 10^{-5}}{1+\frac{k_1k_w}{[\mathrm{H^+}]}+\frac{k_1k_2k_w^2}{[\mathrm{H^+}]^2}+\frac{k_1k_2k_w^3}{[\mathrm{H^+}]^3}} + \frac{1.7}{1+\frac{[\mathrm{H^+}]}{k_1k_w}+\frac{k_2k_w}{[\mathrm{H^+}]}+\frac{k_2k_3k_w^2}{[\mathrm{H^+}]^2}} + \frac{4.3\cdot 10^5}{1+\frac{k_3k_w}{[\mathrm{H^+}]}+\frac{[\mathrm{H^+}]}{k_2k_w}+\frac{[\mathrm{H^+}]^2}{k_1k_2k_w^2}} + \frac{4.3\cdot 10^5}{1+\frac{[\mathrm{H^+}]}{k_3k_w}+\frac{[\mathrm{H^+}]^2}{k_2k_3k_w^2}+\frac{[\mathrm{H^+}]^3}{k_1k_2k_3k_w^3}}$$

$$\text{where}\, k_1 = 10^{4.5}, k_2 = 10^{2.93}, k_3 = 10^{3.57}, k_w = 10^{-14}$$

$$\frac{d}{dt}[\mathrm{Fe^{2+}}] = r_{\mathrm{Fe3+}}[\mathrm{involutin}][\mathrm{Fe^{3+}}] - o_{\mathrm{Fe2+}}([\mathrm{H^+}])[\mathrm{Fe^{2+}}] - radicals$$

$$\frac{d}{dt}[\mathrm{Fe^{3+}}] = -r_{\mathrm{Fe3+}}[\mathrm{involutin}][\mathrm{Fe^{3+}}] + o_{\mathrm{Fe2+}}([\mathrm{H^+}])[\mathrm{Fe^{2+}}] + radicals$$

$$\frac{d}{dt}[\mathrm{involutin}] = p_{\mathrm{inv}}\frac{[\mathrm{Fe^{3+}}]}{[\mathrm{Fe^{3+}}]+k_{\mathrm{invFe3+}}}\frac{[\mathrm{N_{intracellular}}]}{[\mathrm{N_{intracellular}}]+k_{\mathrm{invNic}}} - r_{\mathrm{Fe3+}}[\mathrm{involutin}][\mathrm{Fe^{3+}}]$$

$$\frac{d}{dt}[\mathrm{umb.}] = b_{\mathrm{coum}} + e_{\mathrm{coum}}\mathrm{radicals}$$

$$\frac{d}{dt}[\mathrm{N_{intracellular}}] = u_{\mathrm{NH4+}}[\mathrm{NH_4^+}] + e_{\mathrm{AA}}d_{\mathrm{AA}}[\mathrm{AA}] - d_{\mathrm{Nic}}[\mathrm{N_{intracellular}}]$$

$$fb = ox_{\mathrm{Fb}}[\mathrm{BSAox}] + [\mathrm{BSA}]$$

$$\frac{d}{dt}[\mathrm{H_2O_2}] = p_{\mathrm{H2O2}}\frac{[\mathrm{Fe^{2+}}]^{h_{\mathrm{Fe2+}}}}{[\mathrm{Fe^{2+}}]^{h_{\mathrm{Fe2+}}}+k_{\mathrm{Fe2+}}^{\ \ h_{\mathrm{Fe2+}}}}\frac{[\mathrm{N_{intracellular}}]^{h_{\mathrm{Nic}}}}{[\mathrm{N_{intracellular}}]^{h_{\mathrm{Nic}}}+k_{\mathrm{Nic}}^{\ \ h_{\mathrm{Nic}}}}\frac{fb^{h_{\mathrm{fb}}}}{fb^{h_{\mathrm{fb}}}+k_{\mathrm{fb}}^{\ \ h_{\mathrm{fb}}}}\frac{k_{\mathrm{NH4+}}^{\ \ h_{\mathrm{NH4+}}}}{[\mathrm{NH_4^+}]^{h_{\mathrm{NH4+}}}+k_{\mathrm{NH4+}}^{\ \ h_{\mathrm{NH4+}}}} - r_{\mathrm{Fenton}}[\mathrm{Fe^{2+}}][\mathrm{H_2O_2}][\mathrm{Fe^{3+}}]$$

$$\frac{d}{dt}[\mathrm{BSA}] = -m_{\mathrm{BSA}}radicals[\mathrm{BSA}] - proteases(t)ps_{\mathrm{BSA}}[\mathrm{BSA}] - r_{\mathrm{NH4+BSA}}radicals[\mathrm{BSA}]$$

$$\frac{d}{dt}[\mathrm{BSAox}] = m_{\mathrm{BSA}}radicals[\mathrm{BSA}] - proteases(t)ps_{\mathrm{BSA}}ps_{\mathrm{ox}}[\mathrm{BSAox}]$$

$$\frac{d}{dt}[\mathrm{AA}] = proteases(t)ps_{\mathrm{BSA}}([\mathrm{BSA}] + ps_{\mathrm{ox}}[\mathrm{BSAox}]) - d_{\mathrm{AA}}[\mathrm{AA}]$$

The fitting of parameter values is described later in this text. The observed distributions of fitted parameter values are given in Supporting Information Fig. S4.

The [NH_4^+] were observed to fall exponentially (Fig. 1b), with a rate constant u_{NH4+} = 0.034 h^{-1}. This was accompanied by a drop in pH as the fungal cells exchanged NH_4^+ ions for H^+ (Supporting Information Fig. S1). Since the experimental data also revealed a steady decline in pH in the absence of NH_4^+, the increase in [H^+] was modeled as the sum of a constant (b_{H+} = 3.3 μM h^{-1}) and a term proportional to the uptake of NH_4^+. According to the model, effectively ca. e_{H+} = 0.78 H^+ remained in the growth medium for every NH_4^+ taken up by the fungus.

P. involutus produces substantial amounts of the secondary metabolite involutin, which is thought to be a major player in the reduction of Fe^{3+} to Fe^{2+} (Shah *et al.*, 2015). For the sake of simplicity, the model assumed that all reduction of Fe^{3+} is due to involutin. The measurements of [Fe^{2+}] using the ferrozine assay indicated that nearly all the available Fe^{3+} was reduced within 3–6 d, except when NH_4^+ was excluded from the growth medium (Supporting Information Fig. S3b). The model ascribed this to a strong production of involutin only following a recent N acquisition by the fungus, indicating that the Fenton mechanism only operates when the fungal cells have an adequate intracellular [N]. In the model, this aspect was incorporated in the variable $N_{intracellular}$, which trailed the [NH_4^+] (see below). Since the addition of Fe^{3+} to the culture filtrate did not increase [Fe^{2+}], it appears that excess involutin was not being produced (data not shown). It was thus assumed that the synthesis rate of involutin is proportional to [Fe^{3+}] but limited by the production capacity of the fungus at higher [Fe^{3+}].

Reduction of Fe^{3+} by involutin was assumed to follow mass action kinetics, and its rate was set as constant, $r_{Fe3+} = 1$, as the concentration of involutin could still vary freely through the production rate constant p_{inv}. Oxidation of Fe^{2+} by O_2 was assumed to occur at the same pH-dependent rate as in an aqueous solution, $o_{Fe2+}([H+])$ (Morgan & Lahav, 2007). When H_2O_2 was produced, the primary mechanism by which Fe^{2+} was oxidized in the model was the Fenton reaction (radicals in the equations, i.e., $^{\bullet}OH$). Because of the extensive reduction of Fe^{3+} by involutin, it appeared that the oxidation of Fe^{2+} by H_2O_2 was too subtle to be observed. Oxidation of involutin accompanied the reduction of Fe^{3+} in the model, whereas any recycling of involutin was assumed to already be incorporated in the synthesis term described above.

The production of $^{\bullet}OH$ resulting from the Fenton reaction was measured using coumarin as a probe, which is oxidized by $^{\bullet}OH$ to the fluorescent molecule umbelliferone. The fluorescence signal increased linearly under all conditions (Fig. 1a,b,d), so the production of umbelliferone was modeled with a constant base rate (ca. $b_{coum} = 0.365$ nM h^{-1}) and a rate that depended on an induced $^{\bullet}OH$ production.

The profiles of [Fe^{2+}] and [umbelliferone] in the experiments ruled out the possibility that the timing of the Fenton reaction was controlled through the reduction of Fe^{3+}. The [Fe^{2+}] increased almost immediately in the media (except in the absence of NH_4Cl), but no induction in

the umbelliferone production was observed until day 3 (Supporting Information Fig. S2 and Fig. 1a,b,d). Hence, we concluded that the most direct factor controlling the production of $^{\bullet}OH$ was the regulation of H_2O_2 production.

As described in the main text, an increase in [$^{\bullet}OH$] was observed only when BSA was present and [NH_4^+] became limiting, but this did not occur when BSA was added during prolonged N-starvation (data not shown). One may therefore hypothesize that an activation of the Fenton reaction requires an adequate intracellular [N]. Such regulation may indicate that commitment to the production of H_2O_2 demands sufficient stores of intracellular N, or it may merely reflect the conditions under which such commitment is likely to be profitable for N-acquisition in nature. The intracellular [N] was represented by the variable $N_{intracellular}$, which trailed [NH_4^+] and had a similar decay rate to that of [NH_4^+] (ca. d_{Nic} = 0.036 h^{-1}). In anticipation that N-sources other than NH_4^+ also contributed to [$N_{intracellular}$], a term corresponding to the uptake of N from amino acids (AA) was included. The size of this contribution to [$N_{intracellular}$] was uncertain, with the efficiency parameter e_{AA} mostly in the range of 20–60%.

Induction of $^{\bullet}OH$ production occurred when $N_{intracellular}$ was adequate but the [NH_4^+] was limiting, but only with co-occurrence of high [Fe^{2+}] and a suitable organic substrate, i.e., BSA. As discussed below, considerable effort was dedicated to testing different strategies to model the input from BSA. The detection of BSA by the fungus likely depends on its enzymatic degradation and on subsequent assimilation of amino acids and peptides. Indeed, when the extracellular protease activity was inhibited by pepstatin A, the production of umbelliferone followed the base rate, as if BSA was absent (Fig. 1d). However, extracellular protease activity was not measurably high until about day 4, i.e., 1 d after the induction of $^{\bullet}OH$ production (Fig. 1d). It was thus assumed that a low concentration of protease activity was always present and responsible for the detection of BSA.

Following these considerations, the four putative regulators were combined into a product of four Hill equations, with Fe^{2+}, $N_{intracellular}$, and BSA required for activation, and with NH_4^+ acting as a repressor of H_2O_2 production. Together, these four inputs shape the regulation of $^{\bullet}OH$ production into a Fenton burst. The term "Fenton burst" was chosen since the production of $^{\bullet}OH$ appeared to occur in a burst from day 3 to day 7, in anticipation of, but overlapping with,

extracellular protease activity.

Extracellular protease activity was not measurably high until ca. day 4, a day after the induction of $^{\bullet}OH$ production. Based on the current understanding of the brown rot decay mechanisms (Hatakka & Hammel, 2010), it was assumed that the oxidative degradation does not serve to replace the enzymatic degradation of proteins but, rather, to facilitate it. Thus, the model represents not only the [N] bound in BSA, written as [BSA] for convenience, but also of [N] in oxidized protein fragments, i.e., [BSA_{ox}]. In addition to the oxidation of BSA to BSA_{ox}, both fractions are degraded into amino acids, by extracellular proteases. In the absence of a satisfactory model for the observed protease activity, the protease activity data were fed into the model via the time-dependent function *proteases*(t). The parameter governing the increase of proteolysis rate after BSA oxidation was consistently maximized (ps_{Ox} = 100) (Supporting Information Fig. S5), indicating at least a hundred-fold increase in proteolysis rate of BSA after oxidation with $^{\bullet}OH$ compared to non-oxidized BSA.

The release of NH_4^+ from BSA as a possible outcome of oxidation was also incorporated into the model. Low fitted values for the parameter $r_{NH4+BSA}$ (Supporting Information Fig. S5) suggested that the release of NH_4^+ from BSA accounted for a relatively small fraction of the N acquired by the fungus, which was consistent with [NH_4^+] being at or below the detection limit of ca. 0.15 mM for the last 4 d of the experiments. However, assuming a constant rate for NH_4^+ uptake (which was not necessarily the case), these [NH_4^+] were consistent with an uptake of as much as 10–15% of the N-content of BSA via this mechanism.

Several versions of the model as hitherto described did not reproduce the correct timing and gradual nature of the induction of $^{\bullet}OH$ production. Instead, the Fenton reaction would start abruptly and a full day too late, in response to [NH_4^+] dropping below an unrealistically sharply defined threshold concentration. Several possible feedback mechanisms that could have produced a moderately gradual response were investigated, e.g., regulation of H_2O_2 production by amino acids. The simulations were most consistent with the experimental observations if the feedback was coupled to the oxidized form of BSA. This part of the model may be interpreted to indicate that the aforementioned pathway of BSA detection by low-concentration fungal proteases is boosted when BSA is oxidized, leading to a gradually increasing response to the

presence of BSA and an amplification of the effect of decreasing [NH_4^+].

Model fitting

The model, as described, encompasses equations for 11 variables with 27 parameters. Three of these parameters (u_{HN4+}, b_{H+}, and r_{Fe3+}) were fixed, as described above. In addition, three parameters represented unknown initial concentrations. The parameters were fitted by simulated annealing, as described below. The parameter limits were established based on physical considerations and tentative manual fitting to the empirical data.

The NH_4^+ uptake rate was fitted first, using data for [NH_4^+] until day 4, after which the [NH_4^+] were below the detection limit. The model was then fitted to data for pH, [Fe^{2+}], and umbelliferone fluorescence collected in triplicate at 24-h intervals for 7 d in nine different growth experiments. The 36 time-courses were weighted to equal importance, but with umbelliferone weighted three times higher than [H^+] and [Fe^{2+}] due to its more central role in the model.

Two parameters were devoted to resolving issues with initial [Fe^{3+}]. In the medium with no organic substrate, Fe^{3+} hydrolyzed, aggregated, and was largely removed during the filtration step, with a resulting [Fe^{3+}] of about 18 μM according to the fitted parameters. In growth experiments with low [Fe^{3+}] (0.74 μM Fe^{3+} instead of 74.0 μM), the measurements of [Fe^{2+}] revealed that a substantial amount of Fe^{3+}, corresponding to about 21 μM, must have been carried over from the previous growth medium, presumably adsorbed to the fungal hyphae. This additional amount was modeled in all experimental conditions.

Because of the large number of free parameters and unconstrained variables, no single fitted parameter set was expected to provide reliable predictions. Instead, an ensemble of 80 near-optimal parameter sets was generated, defined as good local optima of a least squares cost function. First, 10^4 random points in parameter space were evaluated, and the 100 best points were used in ca. $1.5 \cdot 10^5$ steps of simulated annealing to optimize the parameters, followed by fine-tuning by local optimization. Running in parallel, the optimization took about 2–4 d to complete using a C++ program described previously (Fogelmark & Troein, 2014). Of the 100 optimized parameter sets, the 80 best sets were retained and used to generate the figures.

As is shown in Fig. 2b-d and Supporting Information Fig. S4, the different parameter sets

have very similar profiles to the measured components, with a somewhat larger variation in the Fenton burst which was not directly constrained by the data. Deviations in the [$^{\bullet}$OH] between experiments and simulations were generally smaller than the experimental variation, and the model was indeed able to explain the patterns observed in the experimental data.

An assessment of the parameter values across the ensemble of 80 fitted parameter sets revealed that parameter determinability for the 27 fitted parameters varied widely (Supporting Information Fig. S5). Some parameters, such as the effect of NH_4^+ uptake on [H^+] and the basal rate of coumarin oxidation (e_{H+} and b_{coum}) had clearly defined values determined to within 1%. Several other parameters could take any value in the allowed range, spanning up to 3–5 orders of magnitude. Despite this variability, conclusions could be drawn from some of the fitted parameters. The parameters governing the Fenton burst were particularly interesting. The input from [Fe^{2+}] had a surprisingly consistent Hill coefficient, ca. $h_{Fe2+} = 2$, suggesting a dimer reaction in the signaling pathway. Regulation by the other two activators, $N_{intracellular}$ (h_{Nic}) and the BSA oxidation feedback (h_{fb}), was mostly high nonlinear, which allowed the Fenton burst to have a well-defined beginning and an end, whereas the repression by NH_4^+ (h_{NH4+}) was gradual.

Conclusions

The modeling supports the view that the regulation of the Fenton reaction in *P. involutus* is controlled by a system that relies on the availability of Fe^{2+}, sufficient N in the intracellular pool, and sensitive detection of protein degradation products. A burst of Fenton activity with the empirically determined temporal profile was most easily achieved by a system that incorporated a positive feedback involving the detection of protein degradation products. Such feedback mechanism enables the fungus to begin oxidative degradation, and provides a possible means for the Fenton burst to end.

Supporting Information References

Fogelmark K, Troein C. 2014. Rethinking transcriptional activation in the *Arabidopsis* circadian clock. *Plos Computational Biology* **10:** e1003705.

Hatakka A, Hammel KE. 2010. Fungal biodegradation of lignocellulose. In: Hofrichter M, ed. *Industrial applications*. Heidelberg, Berlin, Germany: Springer-Verlag, 319-340.

He J, Yang XF, Men B, Wang DS. 2016. Interfacial mechanisms of heterogeneous Fenton reactions catalyzed by iron-based materials: A review. *Journal of Environmental Sciences* **39:** 97-109.

Morgan B, Lahav O. 2007. The effect of pH on the kinetics of spontaneous Fe(II) oxidation by O_2 in aqueous solution - basic principles and a simple heuristic description. *Chemosphere* **68:** 2080-2084.

Shah F, Schwenk D, Nicolas C, Persson P, Hoffmeister D, Tunlid A. 2015. Involutin is an Fe^{3+} reductant secreted by the ectomycorrhizal fungus *Paxillus involutus* during Fenton-based decomposition of organic matter. *Applied and Environmental Microbiology* **81:** 8427-8433.
